# Supplementary figures and images for: Towards robust medical machine olfaction: Debiasing GC-MS data enhances prostate cancer diagnosis from urine volatiles
Source: PLoS One. 2025 May 30;20(5):e0314742. doi: 10.1371/journal.pone.0314742 (PMC12124533; doi:10.1371/journal.pone.0314742)

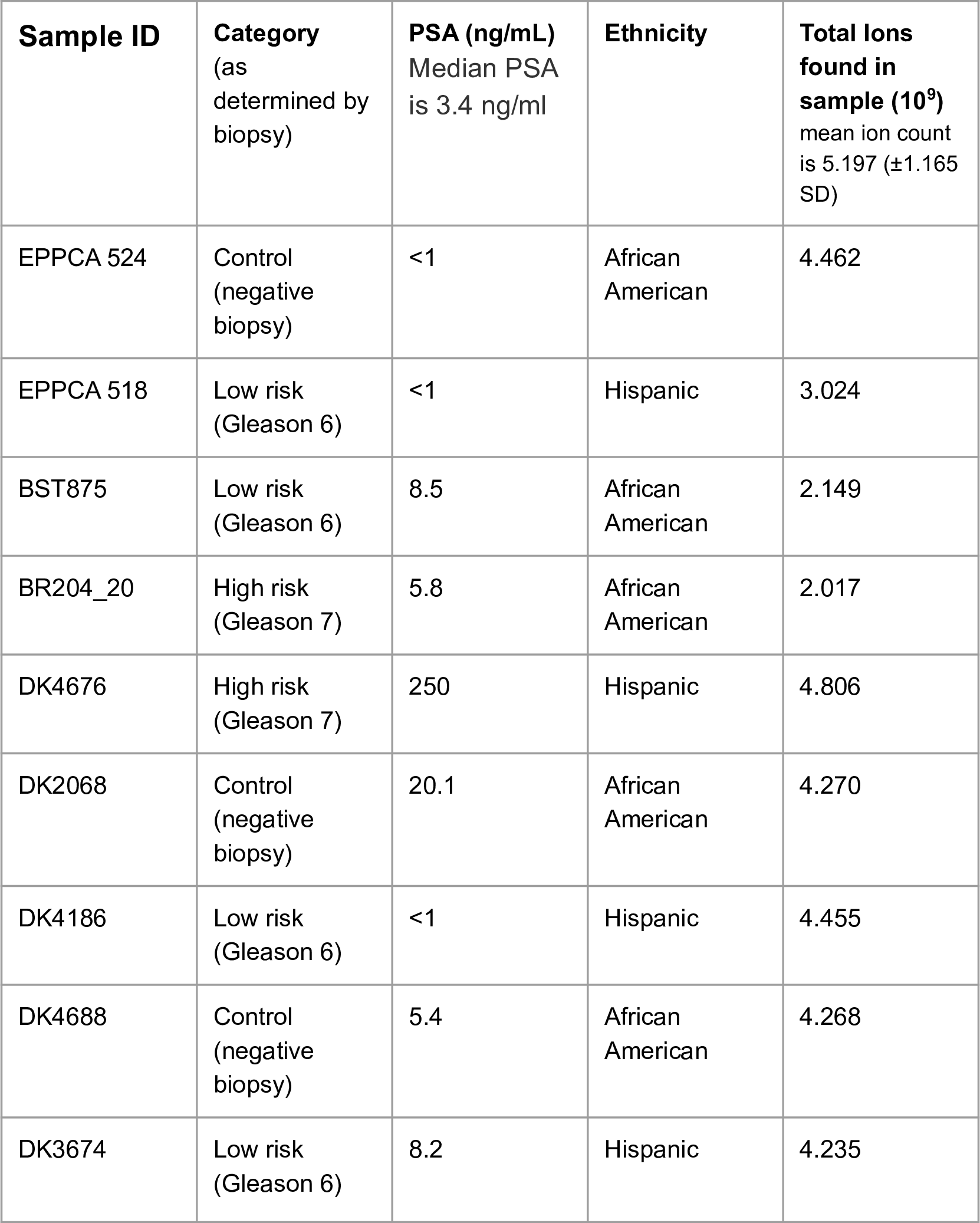

Supplement: S1 Table — Table notes: Each medical center has a different PCa risk distribution; for example, Virginia 1 contains only PCa-positive samples and is therefore highly biased. Overall, 10.1% of the samples come from Massachusetts General Hospital, Boston, Massachusetts, 7.7% come from Duke University Medical Center, Durham, North Carolina, 38.9% come from Michael H. Annabi Internal Medicine Clinic, El Paso, Texas, and 43.3% come from Eastern Virginia Medical Center (batch 1 is 24.4%, sample 2 is 18.9%) (TIF) [file pone.0314742.s001.tif]

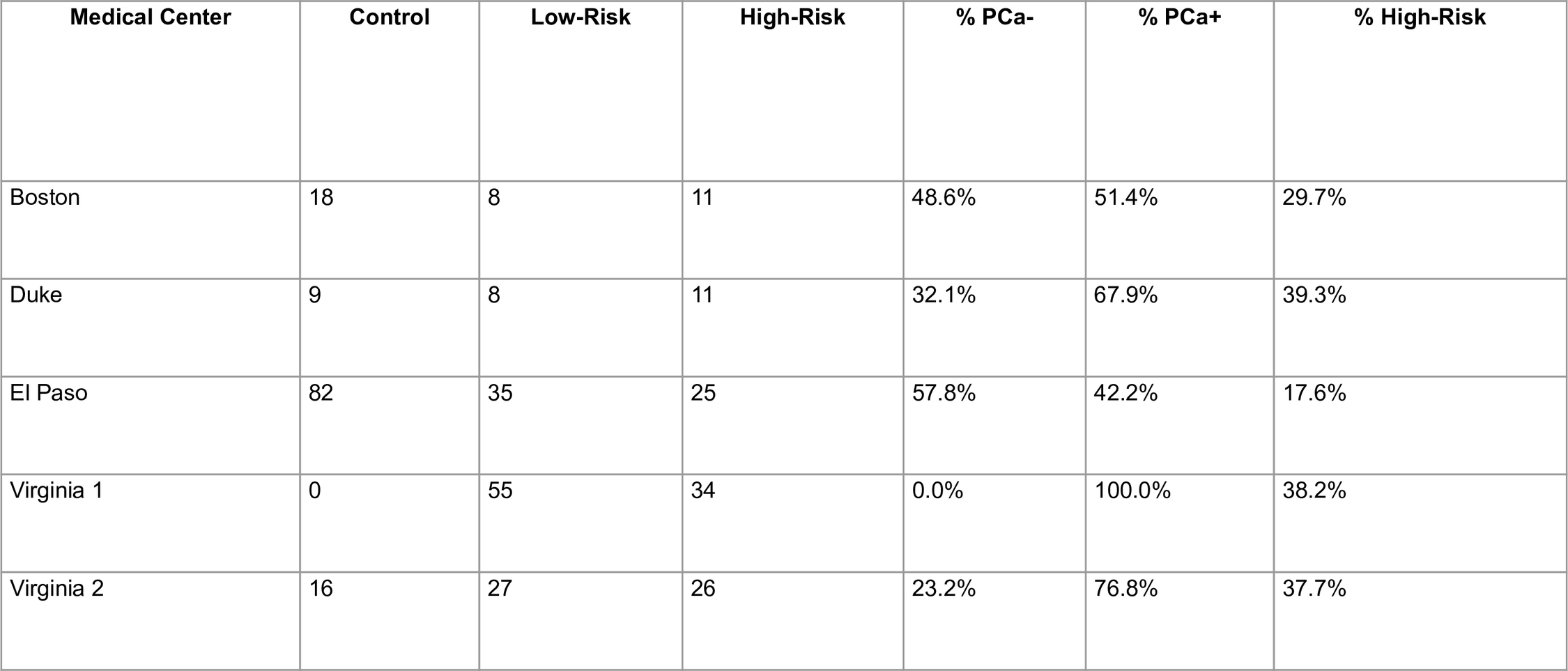

Supplement: S2 Table — The outliers are predominantly from African American and Hispanic heritages, although these groups only represent 55% of the total dataset. Furthermore, six of these outliers have high PSA levels, with two showing exceptionally high values (over the 97th percentile). All samples had lower total ion counts than their batch counterparts. Following batch normalization, the mean ion count was 5.197 (±1.165 SD), with none of the outliers exceeding it. (TIF) [file pone.0314742.s002.tif]

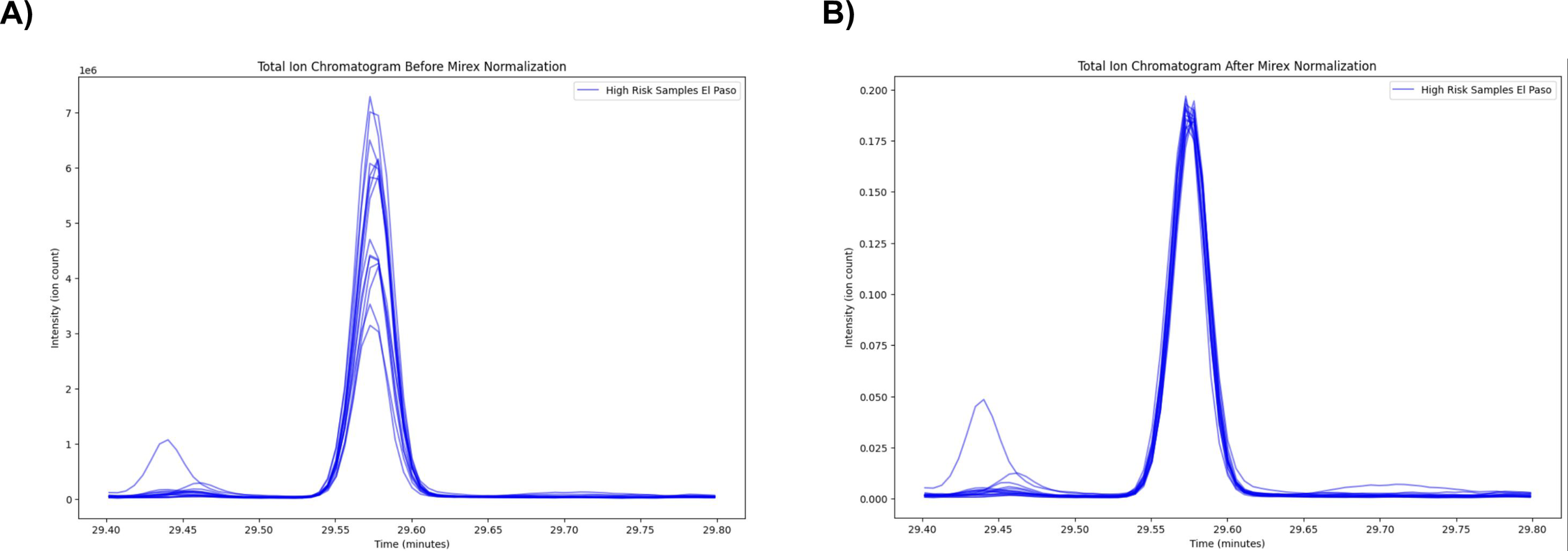

Supplement: S1 Fig — A) Total ion chromatogram of 15 high-risk samples from El Paso, between 29.4 and 29.8 minutes, showing the internal standard Mirex compound peaks. Variation in peak areas indicates the need for normalization. B) Total ion chromatogram of 15 high-risk samples from El Paso, between 29.4 and 29.8 minutes, after Mirex normalization to ensure sample comparability. (TIF) [file pone.0314742.s003.tif]

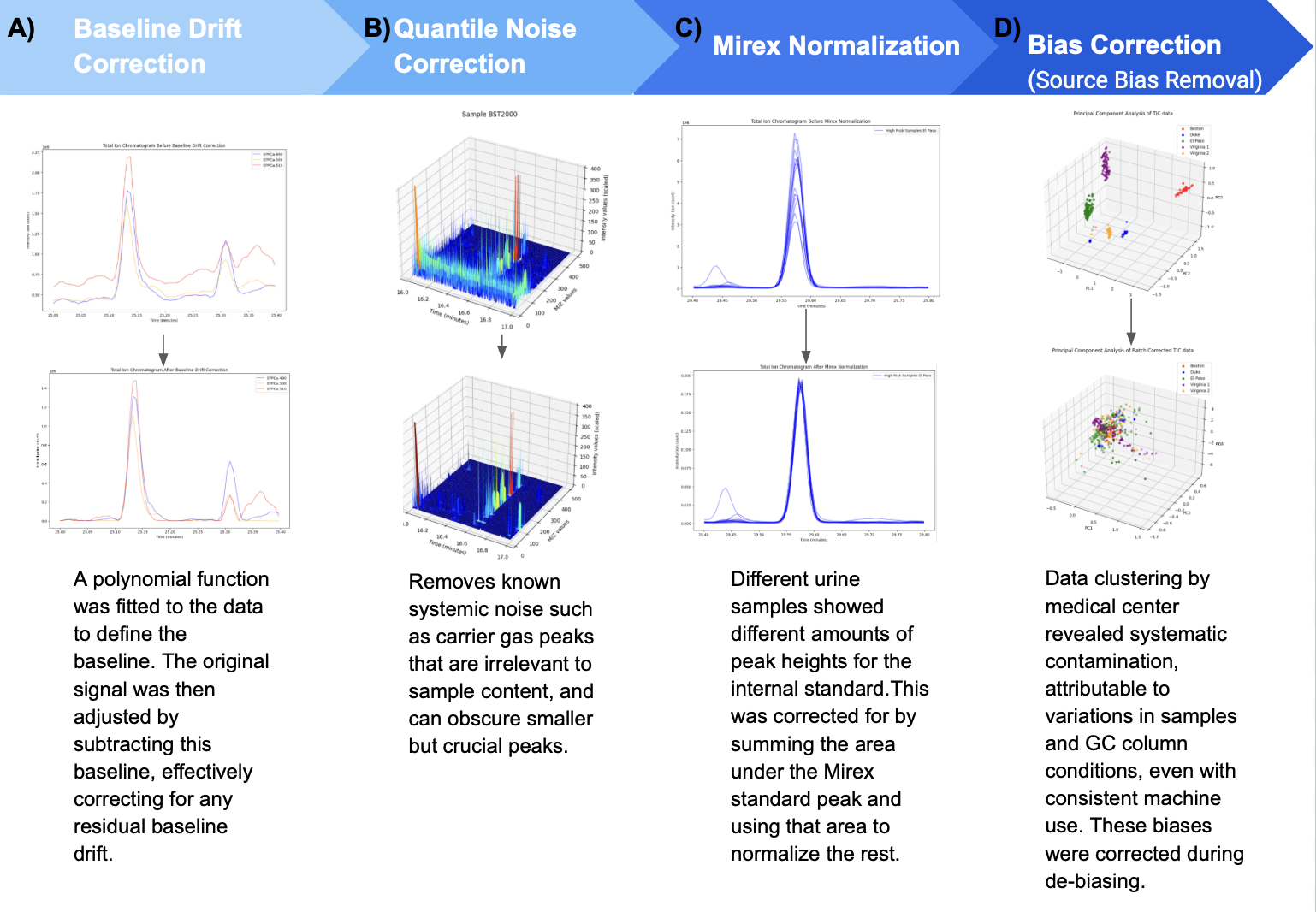

Supplement: S2 Fig — A) Baseline Drift Correction B) Quantile Noise Correction C) Mirex Normalization D) Bias correction (Source Bias Removal). (TIF) [file pone.0314742.s004.tif]
