# Supplementary material for: Towards robust medical machine olfaction: Debiasing GC-MS data enhances prostate cancer diagnosis from urine volatiles
Source: PLoS One. 2025 May 30;20(5):e0314742. doi: 10.1371/journal.pone.0314742 (PMC12124533; doi:10.1371/journal.pone.0314742)
Supplement: S1 Appendix — (PDF) [file pone.0314742.s005.pdf]

Dataset Characteristics

A total of 387 urine samples were initially collected from five different hospitals in the USA, namely: Duke University Medical Center, Durham, North Carolina; Eastern Virginia Medical Center, Norfolk, Virginia; Michael H. Annabi Internal Medicine Clinic, El Paso, Texas, and Massachusetts General Hospital, Boston, Massachusetts. GC-MS TICs were obtained but only 365 patients' samples were selected for use after we rejected TICs with corrupted data. The dataset contains measurements of volatile organic compounds (VOCs) emitted from the patient's urine when heated, measured by Gas chromatography/mass spectrometry coupled with thermal desorption. Each sample consists of ion intensity measurements across 480 mass-to-charge (m/z) ratios for a 41-minutes long period. This 41-minute period was measured using around 7,300 different time points, resulting in a data collection frequency of 3HZ. The dataset is further annotated into three risk categories based on the Gleason score:

- Control: Including samples from patients with Benign Prostatic Hyperplasia (BPH) and those biopsy-proven negative for prostate cancer. 125 total, of which 18 are BPH.
- Low-Risk: biopsy-proven patients with a Gleason score of 6. 133 total.
- High-Risk: Biopsy proven Includes patients with Gleason scores of 7, 8, and 9. 107 total.
