# Supplementary material for: Towards robust medical machine olfaction: Debiasing GC-MS data enhances prostate cancer diagnosis from urine volatiles
Source: PLoS One. 2025 May 30;20(5):e0314742. doi: 10.1371/journal.pone.0314742 (PMC12124533; doi:10.1371/journal.pone.0314742)
Supplement: S2 Appendix — (PDF) [file pone.0314742.s006.pdf]

Hyperparameter Tuning and Model Configuration All models were implemented in PyTorch (version 2.5.1) and trained on a Nvidia Tesla T4 GPU on Google Cloud with Python version 3.11.11.

Grid Search

We explored learning rates of $\{1 \times 10^{-4}, 5 \times 10^{-4}, 1 \times 10^{-3}, 5 \times 10^{-3}\}$ and batch sizes of $\{2, 4, 8, 16\}$, guided by 5-fold cross-validation on the training set. The final configuration chosen was:

- Learning Rate: 1×10^{-4}
- Batch Size: 4
- Optimizer: Adam (default PyTorch values $\beta_1 = 0.9$, $\beta_2 = 0.999$)
- Number of Epochs: We typically trained for 20 epochs but employed early stopping if validation loss did not improve for 5 epochs.

Debiasing Parameter

For the integrated debiasing step, we performed a simple grid search over $\lambda \in \{0.1, 0.3, 0.5, 0.7, 0.9\}$ and selected $\lambda = 0.7$ based on minimizing the combined validation losses. This value provided a good balance between classification performance and source-bias removal.

Cross-Validation Procedure

Each candidate configuration was evaluated using stratified 5-fold cross-validation. We tracked both overall accuracy and balanced metrics (macro-averaged F1), then chose the best-performing model on the held-out folds. Finally, we re-trained with the chosen hyperparameters on the entire training set.
