# Supplementary material for: Towards robust medical machine olfaction: Debiasing GC-MS data enhances prostate cancer diagnosis from urine volatiles
Source: PLoS One. 2025 May 30;20(5):e0314742. doi: 10.1371/journal.pone.0314742 (PMC12124533; doi:10.1371/journal.pone.0314742)
Supplement: Attachment — Submitted filename: #2 PLoS One Debiaser GC-MS rebuttal letter.pdf [file pone.0314742.s008.pdf]

RealNose.ai
UNLOCKING THE SUPERPOWER OF SCENT

Andreas Mershin, PhD *(CSO)*
mershin@realnose.ai

Adan Rotteveel, *(Head of AI)*
rotteveel@realnose.ai

626 Massachusetts Ave
2nd Floor, Unit 8
Arlington, MA 02476 USA
www.RealNose.ai

Dear Dr. Li,

Thank you for your careful review of our manuscript. In response to your comments regarding the corrections needed in order to publish it: specifically for the erroneously referenced papers, we now have made the following revisions:

1. **James et al. (2024)**:
   We have updated the reference list to include the erratum concerning the minor figure correction. In the text, we now cite both the original article and the erratum to ensure full transparency.
2. **Luedemann et al. (2022):**
   Recognizing the correction issued in 2023, we have updated the reference list and updated the citation.
3. **Salinas et al. (2024):**
   For this paper, we have also recognized the erratum, and updated both the reference list and updated the citation.
4. Removed a duplicate reference (from Yang et al)
5. As reviewer # 4 asked we have also combed through the text for sentences such as the one they highlighted to make sure they are optimized.

Thank you all again, we are eager to see our work published as open-access material for all to see in PLoS One

On behalf of the co-authors and with much appreciation for you, the reviewers, and PLoS One,

Adan Rotteveel (corresponding author)

Andreas Mershin, PhD (senior author)
